# Exercise and Sport Performance with Low Doses of Caffeine

**DOI:** 10.1007/s40279-014-0257-8

**Published:** 2014-10-30

**Authors:** Lawrence L. Spriet

**Affiliations:** Department of Human Health and Nutritional Sciences, University of Guelph, Guelph, ON N1G 2W1 Canada

## Abstract

Caffeine is a popular work-enhancing supplement that has been actively researched since the 1970s. The majority of research has examined the effects of moderate to high caffeine doses (5–13 mg/kg body mass) on exercise and sport. These caffeine doses have profound effects on the responses to exercise at the whole-body level and are associated with variable results and some undesirable side effects. Low doses of caffeine (<3 mg/kg body mass, ~200 mg) are also ergogenic in some exercise and sport situations, although this has been less well studied. Lower caffeine doses (1) do not alter the peripheral whole-body responses to exercise; (2) improve vigilance, alertness, and mood and cognitive processes during and after exercise; and (3) are associated with few, if any, side effects. Therefore, the ergogenic effect of low caffeine doses appears to result from alterations in the central nervous system. However, several aspects of consuming low doses of caffeine remain unresolved and suffer from a paucity of research, including the potential effects on high-intensity sprint and burst activities. The responses to low doses of caffeine are also variable and athletes need to determine whether the ingestion of ~200 mg of caffeine before and/or during training and competitions is ergogenic on an individual basis.

## Introduction

The world of sport has always had a high tolerance for the use of caffeine. For many years, caffeine use in sport was restricted or controlled, but athletes were still allowed high urinary caffeine levels (12 μg/mL), before it was considered illegal. Research in the laboratory had determined that athletes needed to ingest ~10–13 mg caffeine/kg body mass (bm) in a fairly short period of time to reach this limit [1, 2]. For a 70 kg person, this amounted to the ingestion of 700–900 mg or the equivalent of ~5–7 cups of coffee, which exceeds typical caffeine use in the general population.

While studies using these high doses of caffeine reported ergogenic effects in endurance-type activities, there were also pronounced effects on the physiological responses to exercise, including increased heart rates, a doubling of the catecholamine levels, higher blood lactate levels, and increased blood free fatty acid (FFA) and glycerol levels in many subjects [1–4]. The ingestion of high caffeine doses also produced troubling side effects of gastrointestinal upset, nervousness, mental confusion, inability to focus, and disturbed sleeping in some subjects, especially those who were habitual light caffeine users [2].

When the caffeine dose was reduced to a moderate level (5–6 mg/kg bm), the ergogenic effects were maintained and the physiological responses and side effects were also reduced but were still present [1, 2, 5]. There have also been many attempts over the years to link these caffeine-induced peripheral physiological responses to the ergogenic benefits of caffeine (see Graham et al. [6] and Spriet [7, 8] for reviews). However, the administration of a low caffeine dose (3 mg/kg bm) also produced an ergogenic effect, with no changes in exercise heart rate and the levels of catecholamines, lactate, FFA, and glycerol [2]. This strongly suggested that the ergogenic effect of caffeine was mediated through the central nervous system (CNS). Previous work demonstrating caffeine’s antagonistic effect on adenosine receptors in the body provided the likely mechanism of action [9–12]. Work with animal models also demonstrated a direct effect of caffeine on the CNS and exercise performance [13].

This paper provides a brief overview of the research that has examined the administration of low doses of caffeine on exercise and sport performance. A low dose of caffeine is defined here as ingesting ~3 mg/kg bm or less, which is ~200 mg of caffeine for a 70 kg individual. This is no more caffeine than may be consumed in 1–2 small cups of coffee or one large coffee. It is ironic that more research has examined the efficacy of low caffeine doses for improving exercise and sport performance in the last 10 years, given that caffeine was removed from the World Anti-Doping Agency list of restricted or banned substances in 2004. It may have been expected that the use of higher caffeine doses would have increased but the majority of research continues to use moderate (5–6 mg/kg bm) or low (≤3 mg/kg bm) doses.

## The Changing Landscape of Caffeine Research

Caffeine research in exercise and sport settings has changed in the past few years. In addition to a greater interest in examining the potential ergogenic effects of low caffeine doses in a variety of situations, research has also examined (1) using time-trial performance tests to simulate real-world situations versus exercise to exhaustion measures; (2) administering divided low doses of caffeine before and during exercise and sport; (3) administering caffeine in alternate forms such as carbohydrate electrolyte solutions (CESs), gels, bars, gums and chocolate; (4) caffeine administration in team-sport settings with sport-specific simulations of performance; (5) the ergogenic effects of caffeine in near-elite and elite athlete populations; and (6) the variable effects of caffeine and the realization that while some generalizations can be made, attention to individual responses and trialing with caffeine ingestion is needed with all athletes.

## Pharmacological Aspects of Caffeine

**Fig. 1:**
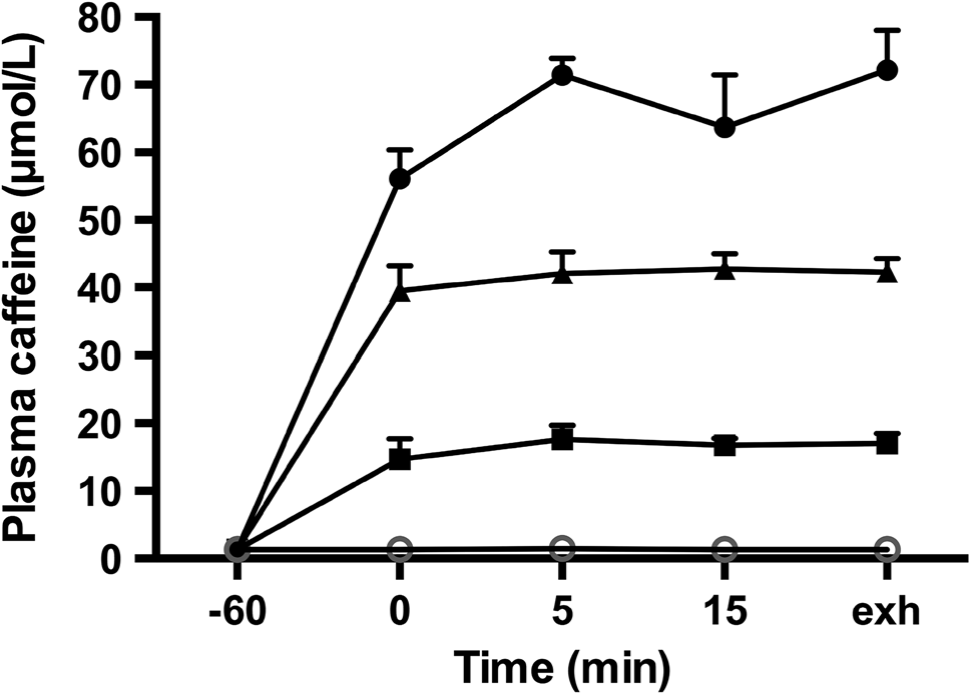
Plasma caffeine concentrations for 1 h at rest and during exercise to exhaustion at 80–85 % of maximum oxygen uptake in recreationally active subjects after the consumption of a placebo (*open circles*), or 3 (*filled squares*), 6 (*filled triangles*) or 9 (*filled circles*) mg/kg body mass of caffeine. Data are means ± standard error (*n* = 8). *exh* exhaustion (reproduced from Graham and Spriet [2], with permission)

Caffeine is rapidly absorbed by the body, when consumed in coffee and capsules, and appears in the blood within 5–15 min and peaks between 40 and 80 min [2, 14–16]. Plasma caffeine levels rise to ~15–20 µmol/L with a low caffeine dose (3 mg/kg bm), ~40 μmol/L with a moderate dose (6 mg/kg bm), and ~60–70 µmol/L with a high dose of 9 mg/kg bm [2] (Fig. 1). Caffeine also has a long half-life (~3–5 h), which makes it well suited to interact with many tissues in the body [16]. However, since caffeine interacts with many tissues, it is difficult to independently study its effects on the CNS, the peripheral nervous system, and the many metabolic tissues in the body (skeletal muscle, liver, heart, and adipose tissue) at rest and during exercise. However, it has been shown that the plasma caffeine levels needed to affect changes in the metabolic tissues are substantially higher than required to affect the adenosine receptors in the brain and peripheral nervous system [9, 11], making it unlikely that there could be major ergogenic effects with caffeine doses of ~3 mg/kg bm or less where plasma levels are 15–20 µmol/L. The lack of changes in heart rate and levels of catecholamines, lactate, FFA and glycerol with this low dose of caffeine supports this argument.

## Are Low Doses of Caffeine Ergogenic During Endurance Exercise?

The interest in caffeine as a potential ergogenic aid during endurance exercise began in large part due to the research from David Costill’s laboratory in the late 1970s. Trained cyclists improved their ride times to exhaustion at ~80 % of maximal oxygen consumption (*V*O_2max_), from 75 min in the placebo condition to 96 min following the ingestion of 330 mg of caffeine in coffee [17]. This caffeine dose was not low at ~5 mg/kg bm, but a second study gave only 250 mg of caffeine at the beginning of exercise and then another 250 mg in seven doses during exercise and reported a 20 % increase in the work completed during 2 h of cycling [18].

### Laboratory-Based Endurance Cycling Studies

**Fig. 2:**
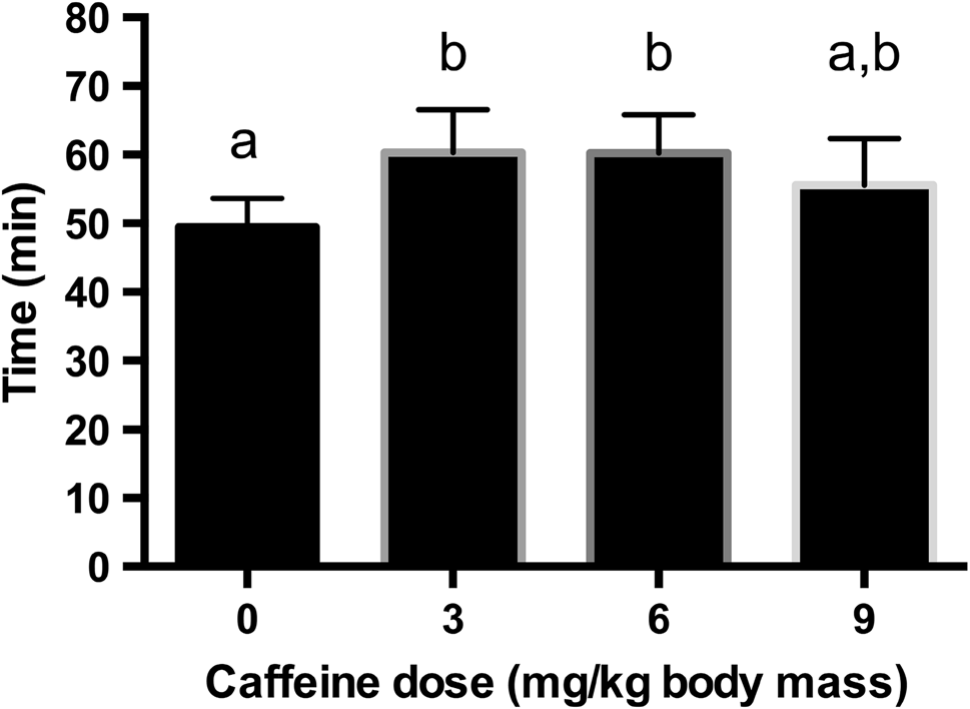
Effects of ingesting no caffeine (0) or 3, 6 or 9 mg/kg body mass of caffeine (dose) on running time to exhaustion at ~85 % of maximum oxygen uptake. Data are means ± standard error (*n* = 8). *Bars* *with different letters* are significantly different, and *bars with the same letters* are not significantly different (reproduced from Graham and Spriet [2], with permission)

This early work hinted that there may be an ergogenic effect of low doses of caffeine but it was not until 1995 that a dose response study examined the effects of 3, 6 and 9 mg/kg bm on the performance of well-trained runners [2]. Subjects abstained from caffeine use for 48 h, then consumed a random dose of caffeine or placebo in capsule form 1 h before running to exhaustion at ~85 % *V*O_2max_ on a treadmill in ambient laboratory conditions on four separate occasions. Endurance performance was enhanced by 22 % over the placebo run of 49.4 ± 4.2 min (mean ± standard error [SE]) following the ingestion of 3 and 6 mg/kg bm caffeine, but only by 11 % and non-significantly following the highest caffeine dose (Fig. 2).

A later study examined the effects of two low caffeine doses and one moderate caffeine dose given with a CES on the ability to complete a set amount of work that required ~1 h [19]. Well-trained cyclists and triathletes randomly received either a placebo or a caffeine dose of 2.1, 3.2 or 4.5 mg/kg bm. The caffeine doses were partitioned with ~60 % of the dose administered ~20 min before exercise, 20 % after 20 min of cycling, and the remaining 20 % at 40 min into the ride. The time-trial performance was 62.5 ± 1.3 min (mean ± SE) in the placebo trial and sequentially decreased with increasing caffeine doses to 61.5 ± 1.1, 60.4 ± 1.0 and 58.9 ± 1.2 min. Therefore, while the low doses of caffeine were performance-enhancing, this study suggested that the moderate dose (4.5 mg/kg bm) was the most ergogenic, unlike the results reported by Graham and Spriet [2] where no differences were reported between 3 and 6 mg/kg bm.

### Low Caffeine Doses Given Late in Prolonged Endurance Exercise

An interesting study was published in 2002 suggesting that well-trained athletes are very sensitive to small doses of caffeine late in prolonged exercise without taking any caffeine before exercise [20]. Scientists at the Australian Institute of Sport were aware that endurance cyclists preferred to switch from a CES to flat cola in the later stages of 2- to 6-h road races. They then tested whether this practice was ergogenic and whether the active ingredient(s) in the cola was the extra carbohydrate (CHO) [~11 % CHO vs. ~6 % in the CES] or the caffeine. Eight well-trained cyclists completed four double-blinded and random trials where they cycled for 102 min at ~70 % *V*O_2max_, followed by a time trial where they completed 7 kJ/kg bm as fast as possible (~27 min). The subjects were mild caffeine users (>150 mg/day) and abstained from caffeine use for 48 h before every trial. In all trials, they consumed 5 mL/kg bm of CES at 20, 40 and 60 min. At 80 and 100 min (and 120 min, if desired) they received one of four subgroups: CONTROL (decaffeinated cola, 6 % CHO), CAFFEINE (90 mg caffeine +, 6 % CHO), Extra CHO (decaffeinated cola, 11 % CHO), and COKE (90 mg caffeine +, 11 % cola). Performance times for CONTROL and CAFFEINE were 27:05 ± 0:42 (min ± sec; mean ± SE) and 26:36 ± 0:42 min, and for the Extra CHO and COKE trials were 26:55 ± 0:43 and 26:15 ± 0:43 min. COKE enhanced the time-trial performance by 3.3 % relative to CONTROL, with a 2.2 % time-trial enhancement with CAFFEINE [20]. The authors concluded that ~67 % of the improvement in time-trial performance was the effect of caffeine, with the remaining 33 % due to the additional CHO. The average total caffeine intake at 80, 100 (and for some, 120) min of cycling was only 133 mg or ~1.9 mg/kg bm, resulting in plasma levels of less than 10 µmol/L. These low levels of caffeine intake and plasma accumulation did not affect any of the physiological responses to exercise, suggesting that the beneficial effects of caffeine were manifested in the CNS late in exhaustive exercise.

In a similar study, the effects of two low doses of caffeine on time-trial performance following a prolonged cycle were examined [21]. Fifteen well-trained cyclists and triathletes, who were not caffeine users, completed four trials in a double-blinded and random fashion. They cycled for 120 min at ~60 % *V*O_2max_, with five hill climbs at ~85 % *V*O_2max_, followed by a time trial where they completed 6 kJ/kg bm as fast as possible (~25–30 min). In all trials, subjects consumed 5 mL/kg bm of CES (6 % CHO, 20 mmol/L sodium) throughout the 120 min. At 80 min, subjects received one of four conditions in their CES: placebo (regular CES), CAF100 (100 mg caffeine, ~1.5 mg/kg bm), CAF200 (200 mg caffeine, ~3 mg/kg bm), or a random ‘repeat’ of one of the other three conditions. The ‘repeat’ trial helped complicate the subjects’ perception of what they had received, and post-trial questionnaires confirmed that the ‘double-blinding’ was successful. Subjects completed the time trial in 28:41 ± 0:38 (min ± sec; mean ± SE) in the placebo condition, were significantly faster in the CAF100 (27:36 ± 0:32 min) trial, and faster again in the CAF200 (26:36 ± 0:22 min) trial. Time-trial performance in the ‘repeat’ trials (five subjects completed two placebo trials, five completed two CAF100 trials, and five completed two CAF200 trials) were 27:19 ± 0:30 for the first trial and 27:30 ± 0:35 min for the second trial. Plasma caffeine levels were not measurable in the placebo condition and reached 14.9 μmol/L before (120 min) and 13.8 µM after the time trial in CAF100, and 24.9 and 25.6 µmol/L at the same time points in CAF200. These results demonstrated that two low caffeine doses (~1.5 and ~3 mg/kg bm) were ergogenic in well-trained cyclists when ingested late in an exhaustive ride, during a training-session-ending time trial [21]. The 200 mg dose was more potent than 100 mg of caffeine. There were no differences in the physiological responses during the initial 120 min of submaximal exercise (heart rate, respiratory exchange ratio, and epinephrine, glucose, lactate, glycerol, and FFA levels) and prior to the time trials between the conditions, supporting a CNS mechanism for the improvement in performance.

### Low Caffeine Doses Ingested Before Endurance Exercise

In other recent studies that examined the effects of low doses of caffeine given 1 h before prolonged exercise, improvements in performance have also been shown, but not at all doses. Jenkins et al. [22] gave well-trained cyclists either a placebo or 1, 2 or 3 mg/kg bm caffeine 1 h before exercise in a randomized design. Subjects cycled for 20 min at 80 % *V*O_2max_, completed a 5 min active recovery, and then rode as hard as possible in a 15 min time period. The work done in 15 min was not improved by 1 mg/kg bm caffeine but increased significantly by 4 % and 3 % after 2 and 3 mg/kg bm, respectively. The authors also noted the considerable variability that existed between subjects [22]. In a similar study, Desbrow et al. [23] gave well-trained male cyclists either a placebo or 1.5 or 3 mg/kg bm in a double blind, randomized manner 1 h before exercise. The subjects cycled for 120 min at ~70 % *V*O_2max_, followed by a 7 kJ/kg bm time trial. In this study, there were no improvements in time-trial time performance with caffeine [23].

Irwin et al. [24] published an interesting study that was designed to determine whether a low acute dose of caffeine (3 g/kg bm) was ergogenic for endurance cycling performance following 4 days of caffeine withdrawal or no withdrawal in habitual caffeine users. Well-trained male cyclists completed a series of conditions and trials where they received either a placebo or caffeine 90 min before exercise, following caffeine withdrawal or no withdrawal. The exercise was a 1 h cycle time trial at ~75 % of peak sustainable power output. Following a withdrawal period, performance was improved by 1:49 ± 1:41 min (mean ± SE) or 3 %, and by 2:07 ± 1:28 min or 3.6 % following no withdrawal and these results were not significantly different. In this study, the habitual caffeine-user athletes experienced a significant improvement in performance from ingesting a low caffeine dose 90 min before exercise, regardless of whether they withdrew from caffeine or not [24]. Finally, in a similar study from the same laboratory, Desbrow et al. [25] reported that caffeine doses of 3 and 6 mg/kg bm were ergogenic when well-trained male subjects completed a 1 h cycle time trial at ~75 % of peak sustainable power output compared with placebo. Performance improvements were 4.2 and 2.9 % for the two caffeine doses, prompting the authors to conclude that doubling the caffeine dose from a low to moderate dose did not provide additional ergogenic benefits.

## Are Low Caffeine Doses Ergogenic in Sports?

Much of the experimental work examining the performance effects of caffeine in general has occurred in laboratory settings. While many of these studies have attempted to mimic field settings (cycling, rowing, running, etc.), they are removed from the field-based environment. An excellent review addressing the need for more field- and practical-based caffeine studies with actual sport-specific athletes and performance measures was published by Burke [26] in 2008. A second review stressed the importance of using time-trial performance assessments in laboratory and field studies examining caffeine and performance [27]. The conclusions of the meta-analyses of the studies using time trials gave the same conclusion as earlier studies using time to exhaustion as the performance measure—caffeine is ergogenic [27]. However, researchers are well aware of the difficulty in controlling field studies where environmental and competition characteristics can vary from test-day to test-day, and that mimicking ‘game performance’ can also be difficult either for the single athlete or when the success of a team is dependent on many players. Nonetheless, there have been an increasing number of publications that have assessed the effects of ingesting caffeine in sport settings. and some of these studies have used low doses.

### Running

Wiles et al. [28] published a comprehensive study demonstrating that ~150–200 mg of caffeine ingested as coffee 1 h before exercise improved 1,500 m running performance in well-trained runners. In one experiment, 1,500 m time was improved by 4.2 s with caffeine and, in a second experiment, where the runners ran the first 1,100 m at a constant speed and then self-selected their speed over the final 400 m, running speed was improved over the final lap by 0.6 km/h, which amounted to 10 m. However, in a longer road race, 98 runners completed an 18 km run three times in 8 days in a cool environment and found no effect of a low dose of caffeine on performance [29]. Subjects drank 150 mL of either water, a CES, or a CES with 150 mg/L of caffeine, on four occasions during the races—at the start and after 4.5, 9 and 13.5 km. The total ingested caffeine was 90 mg or ~1.3 mg/kg bm.

More recently, Bridge and Jones [30] reported a 24 s or 1.8 % improvement in 8 km run time on a track with well-trained male runners when ingesting 3 mg/kg bm of caffeine 1 h before racing. Conversely, Schubert et al. [31] reported no performance-enhancing effects of two ‘energy drinks’ containing either 80 or 140 mg caffeine versus a caffeine-free placebo in a 5 km treadmill time trial. However, this study did not control for the various other ingredients that were present in the two energy drinks and therefore was not able to examine the individual effect of caffeine.

### Stop-and-Go Individual and Team Sports

There have been some studies examining the potential ergogenic effects of what might be called ‘stop-and-go’ individual and team sports [26], most of which have not been conducted with low caffeine doses. A study with male collegiate tennis players demonstrated some improvements in forehand shot performance when 3 mg/kg bm was consumed 90 min before a simulated tennis match [32]. Stevenson et al. [33] conducted a comprehensive study of golf putting performance and alertness when comparing an energy free, flavoured drink versus a CES with caffeine. Experienced male golfers drank 5 mL/kg bm of the selected drink before a round of golf and 2.5 mL/kg bm at holes 6 and 12. Putting performance (2 and 5 m) was assessed at each hole and self-rated mood assessments were done every three holes. Subjects consumed a total of 1.6 mg/kg bm caffeine and 0.64 g/kg bm CHO during the round. The CES drink with caffeine improved putting performance and increased feelings of alertness [33]. It should be pointed out that because both caffeine and CHO were consumed, the study design did not allow the individual effect of caffeine to be determined.

Two recent studies examined the effects of ingesting 3 mg/kg/bm caffeine on volleyball performance in females and males [34, 35]. In both studies, the caffeine was administered in an energy drink, with a caffeine-free energy drink serving as the control. The players completed a series of volleyball performance tests and played in a simulated match on both occasions. Ball velocity in a spike test, several jump tests, the time to complete an agility test, and the number of successful volleyball actions during the game all improved in the caffeine trials for both females and males. The authors stressed that both physical performance and the accuracy of the volleyball skills were improved with caffeine ingestion in a commercially available energy drink [34, 35].

Two team-sport studies also suggested that low doses of caffeine may improve aspects of soccer and rugby playing, although the studies gave 3.7 and 4.0 mg/kg bm caffeine, respectively. In the soccer study, players completed two 90 min intermittent shuttle running trials, consuming a CES on one occasion and the same CES with caffeine on the other [36]. The solutions were ingested before the trial and every 15 min throughout. Adding caffeine to the CES improved sprint performance, counter-movement jumping, and offset the fatigue-induced decline in the self-selected components of performance [36], In the rugby study, the players again were given either a placebo, CES, or a CES with caffeine before and during a rugby-union-specific shuttle running protocol [37]. In the CES with caffeine trial, the likelihood of a 2 % improvement was 98 % over placebo and 48 % over CES alone. Motor skills were also performed faster in the CES plus caffeine trial versus the other two trials, and 15 m sprints were faster than placebo. The authors concluded that the co-ingestion of CHO and caffeine was likely to benefit rugby union performance [37]. It would be interesting to repeat aspects of these studies with slightly lower caffeine doses.

## Low Caffeine Doses and High-Intensity Exercise that Relies on Anaerobic Energy Production

Many forms of exercise and sports are reliant on bursts of activities or sprints, where the dominant portion of the energy production must be provided by non-oxidative or ‘anaerobic’ energy sources [38, 39]. Phosphocreatine (PCr) and adenosine triphosphate (ATP) production in the glycolytic pathway are the two main pathways for the production of anaerobic energy. While the PCr store can be consumed in a few seconds and the heavy use of the glycolytic pathway can lead to acidosis in muscle cells, these pathways are able to provide energy very quickly and in large quantities for short periods of time to allow athletes to complete very powerful movements [38, 39]. In many sports, the ability to repeatedly burst or sprint is essential to success. In stop-and-go and power-based sports, these bursts often occur on the back of already high energy production from the aerobic system. The question for this paper is whether this type of activity is improved by caffeine ingestion and specifically low doses of caffeine.

Early reviews generally concluded that there was no benefit of caffeine for burst activities [8, 9] as the majority of studies reported no effects. However, as the number of studies examining this type of work increased, more recent reviews suggested that there were benefits in about 50 % of the published studies in power-based sports, resistance training paradigms, repeated high-intensity intermittent exercise, and isometric and isokinetic muscle force production and endurance [40, 41]. For example, indices of strength in highly resistance-trained males were improved with caffeine ingestion of 7 mg/kg bm [42], and more weight was lifted and a greater peak power was attained in a Wingate test in competitively trained males following the ingestion of 5 mg/kg bm caffeine [43]. Short-term high-intensity cycling lasting about 1 min was also improved by the ingestion of 5 mg/kg bm caffeine in two studies [44, 45]. However, these studies did not measure lower doses of caffeine.

Almost no work exists in this area with low caffeine doses. However, a research group in France reported ergogenic effects of a 250 mg dose of caffeine on work maximum in a test of maximal anaerobic power [46] and in repeated 100 m sprints in trained swimmers [47]. More recently, Astorino et al. [48] examined the effects of ingesting either 2 or 5 mg/kg bm caffeine on isokinetic knee flexion performance and reported no effect of the low caffeine dose on peak torque or knee extension/flexion total work or power. Therefore, the conclusion here must be that very little ‘low-dose’ caffeine research has been done in this area, although it may be argued that this stems from the somewhat equivocal results that have been reported with moderate and high doses of caffeine on burst and sprint activities.

## Low Caffeine Doses and Vigilance, Alertness, Mood and Cognitive Function

There is a large literature that has examined the use of caffeine for maintaining vigilance, alertness, mood, executive control, and related parameters. This research often investigates situations where maintaining vigilance and performance is critical, as in the military and other professions where people are awake for long periods of time. Lieberman and colleagues have worked in this area for many years and published many papers on the potential usefulness of caffeine. For example, they gave 20 subjects four random doses of caffeine (32, 64, 128 and 256 mg) and assessed auditory vigilance and reaction time and mood in healthy volunteers [49]. All four caffeine doses improved the performance of these tests. More recent work indicated that the optimal dose of caffeine is ~200 mg when vigilance, mood, alerting, orienting, and executive control were assessed against other caffeine doses or no caffeine [50, 51]. The plateauing of beneficial effects at ~200 mg of caffeine is believed to match the adenosine-mediated effects on dopamine-rich areas in the human brain and their involvement in the executive control of visual attention and alerting [49]. Hogervorst et al. [52, 53] published two studies suggesting that low doses of caffeine improve cognitive performance during and after strenuous exercise. The first study had trained cyclists/triathletes complete an all-out 1 h cycling time trial on five occasions. They randomly received a water placebo, a CES placebo, or one of three CESs with either, 150, 225 or 320 mg of caffeine before and during the cycle [52]. Cognitive tests, including memory, psychomotor and attention tasks, were carried out before and immediately after the time trial. The two low doses of caffeine improved all cognitive functions following exercise and the higher dose provided no further improvement. These results clearly demonstrated that the ability to concentrate and make decisions was improved by caffeine immediately after exhausting exercise, leading to the suggestion that this effect would also be present in the later stages of exercise [52]. A second study directly measured the effects of ingesting 100 mg of caffeine in an energy bar (with 45 g CHO) before exercise and again at 45 and 115 min of a 3 h cycle at 60 % *V*O_2max_, followed by a time-to-exhaustion trial at 75 % *V*O_2max_ on cognitive function measures [53]. A second trial gave the energy bar only, and a third was a placebo trial with an energy- and caffeine-free beverage. Cognitive function tests (Stroop test and rapid visual information processing tests) were administered before exercise and after 70 and 140 min of the trial and 5 min after the trial. Performance was best following caffeine ingestion (total of 300 mg before and during exercise) on the complex information processing tests, especially after 140 min of cycling. Cycle-ride time to exhaustion was also the longest in the caffeine trial. These results suggest that low-dose caffeine ingestion during prolonged exercise helps with decision making and could be useful for all sports where critical decision making is important for success late in an event or game [53].

## Low Caffeine Doses and Related Issues

### Pain

There is a large body of literature reporting that the perception of pain during moderate and intense exercise is reduced following the ingestion of 5–10 mg/kg bm caffeine in men and women [54–56]. However, not all studies have reported an attenuation of pain during strenuous or high-intensity exercise. Astorino et al. [57] reported no effect of 2 or 5 mg/kg bm caffeine on pain perception during repeated bouts of high-intensity exercise. A second study reported that a 3 mg/kg bm caffeine dose 1 h before exercise and another 3 mg/kg bm dose 45 min into a 2 h submaximal ride, followed by a 15 min performance trial, reduced leg pain by 27 % in a trial in the heat (33 °C) but had no effect in a cool environment (12 °C) [58]. It should be noted that pain perception was 74 % higher in the hot versus cool trial and caffeine was able to partially attenuate this.

### Exercise in the Heat

Exercising in a hot environment is less well tolerated than exercise in a cool environment and several studies have examined whether caffeine may counteract the fatigue of exercise in the heat. The literature is mixed, with some studies reporting that 5, 6 or 9 mg/kg bm caffeine did not improve endurance performance in the heat [59, 60], with others suggesting that 6 mg/kg bm is ergogenic [58, 61]. However, the author is not aware of any studies that have examined the effects of low doses of caffeine in the heat, especially late in exhaustive exercise.

### Gastrointestinal Function

One study examined the effects of low doses of caffeine (95 mg) in a CES on gastrointestinal function given before exercise and at 20 and 40 min into 90 min of cycling at ~70 % *V*O_2max_ [62]. Caffeine (285 mg in total) had no effect on gastric emptying, gastric pH, orocecal transit time, and intestinal permeability. Interestingly, glucose absorption was increased in the caffeine trial. A subsequent study examining the effects of caffeine on glucose absorption also reported an increase but with a high caffeine dose of 10 mg/kg bm during cycling [63], while two other studies reported no effect with 1.5, 3 and 5.3 mg/kg bm [23, 64]. So on balance, it seems unlikely that low doses of caffeine would affect glucose absorption on a consistent basis.

### Immune System

Natural killer (NK) cells comprise about 5–20 % of all lymphocytes in the blood, and represent the first line of defence against infectious agents as they do not need prior sensitization or require specific antigen recognition to attack target cells [65]. They also play an important role in defence against viral infection and tumor immune surveillance [65]. The functional abilities of NK cells decreases after intense exercise, and this decrease may explain why there is a high incidence of viral respiratory infection in elite athletes [66]. The NK cells have A_1_ and A_2_ adenosine receptors, and the exercise-induced decreases in activation may be due to increased adenosine and epinephrine concentrations. With this in mind, and the fact that caffeine is an adenosine receptor antagonist, Fletcher and Bishop [67] examined the effect of moderate (6 mg/kg bm) and low (2 mg/kg bm) doses of caffeine on NK cell function. Endurance-trained males cycled for 90 min at 70 % *V*O_2max_ on three occasions after ingesting no caffeine, the low caffeine dose, or the high dose. Both doses of caffeine increased both the unstimulated, or natural state of NK cell activation, and the antigen-stimulated NK cell activation 1 h after exercise; this response was not dose-dependent. The authors hypothesized that caffeine may have antagonized the adenosine receptors on the NK cells, leading to the increased state of activation following exercise [67].

## Delivery of Low Caffeine Doses in Alternative Forms

Caffeine administration in most research settings is delivered in coffee or in tablets/capsules along with water, where there is a known amount of caffeine ingested. Caffeine is also available in gels and bars and in some sports drinks for use by athletes before and during athletic events. It might be assumed that the appearance of caffeine in the blood would be slightly delayed with gels and bars compared with ingestion in coffee or tablets/capsules ingested with water but this does not appear to have been studied. The study discussed in Sect. 7 in which the caffeine was given in a bar, produced an ergogenic effect, therefore any absorption effects may be small [53].

Another form of delivery that has received some interest is chewing gum. Kamimori et al. [16] compared the rate of absorption of 50, 100 and 200 mg caffeine in chewing gum versus the same doses in capsules in healthy volunteers. All subjects had abstained from caffeine for 20 h and there were 12 subjects in each treatment group. Eight blood samples were taken from 5 to 90 min after caffeine ingestion, and eight more were taken from 2 to 29 h post-administration. The rate of caffeine absorption was significantly faster from the gum versus the capsules, suggesting that absorption from the buccal mucosa in the mouth was a contributing factor when chewing gum was administered [14, 16]. However, over time, the gum and capsule formulations provided essentially the same amounts of caffeine for the 100 and 200 mg doses. If rapid caffeine absorption is required in a sport situation, chewing gum may be the desired form of delivery. A second study demonstrated that plasma caffeine levels can be maintained at desired concentrations with repeated doses (2 h apart) of caffeine delivered in gum [68].

A few studies have examined the potential ergogenic effect of caffeine administration in gum. Ryan et al. [69] administered two pieces of caffeinated chewing gum (200 mg total) at one of three time points, either 35 or 5 min before exercise or 15 min into cycling at 85 % *V*O_2max_ to exhaustion lasting ~30–35 min. A placebo was given at the other two time points and all three points during the control trial. The subjects were college-age, physically active male volunteers. The caffeine in the gum did not improve endurance performance at any of the administration times [69]. In a follow-up study, Ryan et al. [70] gave 300 mg of caffeine in gum either at 120 min, 60 min or 5 min before exercise. In the placebo trial, subjects received no caffeine. The subjects in this study were well-trained cyclists, and the performance task was cycling at 75 % *V*O_2max_ for 15 min, followed by a time trial where 7 kJ/kg bm of work was completed as fast as possible. Caffeine improved cycling performance only in the trial where the caffeine was administered 5 min before exercise compared with the placebo trial [70].

One report examined the effects of caffeine given as chewing gum on repeated cycle sprint ability [71]. Competitive cyclists completed four sets of 30 s maximal sprints, with five sprints/set and each sprint was separated by 30 s of active recovery. Caffeine (240 mg or ~3 mg/kg bm) was administered following the second set and the rate of power output decline in the final two sets (10 sprints) was significantly reduced by the caffeinated gum compared with the placebo group. A second study reported that standing shot-put performance was improved following the administration of 100 mg caffeine in gum [72].

Another emerging area for exposing the body to caffeine is mouth rinsing. The premise is that small volumes of fluid containing high concentrations of caffeine could be mouth-rinsed for 5–10 s periods. While this is unlikely to result in significant caffeine absorption, it would test the possibility that caffeine is sensed in the mouth with signals sent to the CNS. Doering et al. [73] had subjects complete a 1 h cycling time trial on two occasions. They mouth-rinsed for 10 s with 25 mL volumes containing either 35 mg caffeine or no caffeine on eight occasions during the time trials. Time-trial performance was unaffected by the caffeine mouth-rinses and no increase in plasma caffeine was detected. However, an earlier report by Beaven et al. [74] had subjects complete 5 × 6 s sprints with 24 s of active recovery between sprints. Subjects mouth-rinsed with 25 mL volumes containing either a CHO solution, a 1.2 % caffeine solution (~300 mg caffeine) or a non-caloric solution for 5 s just prior to each sprint. The caffeine mouth-rinse improved peak power in the first sprint only. When caffeine and CHO were combined in a second study and compared with CHO only, the caffeinated mouth-rinse condition again improved peak power in the first sprint [74]. The authors did not measure plasma caffeine to see if mouth rinsing with a high caffeine-containing solution resulted in significant absorption of caffeine, and it is not clear why the caffeine effect was only present in the first of five sprints. Further work will be needed to determine whether mouth rinsing with caffeine is ergogenic in the absence of absorption into the blood.

Lastly, there are commercially available products where caffeine can be delivered in aerosol form, with some products claiming to deliver 100 mg of caffeine per spray or squirt of aerosol. There are no published reports measuring the time of caffeine absorption into the blood using these products, and it seems unlikely that these claims are true. However, delivering caffeine in a gas form into the lungs sets up the possibility that rapid absorption could occur. In this case the absorbed caffeine would be delivered straight from the lungs to the heart and this would not be desirable in some cases. An absorption study measuring the effectiveness of these products is needed.

## Conclusions

It has long been known that moderate to high caffeine doses (5–13 mg/kg bm) ingested ~1 h before and during exercise increase endurance exercise performance in laboratory and sport field settings. Recent work also suggests that caffeine is ergogenic in some short-term high-intensity exercise and sport situations and also in team-sport simulations. Lower caffeine doses (≤3 mg/kg bm, ~200 mg) taken before exercise also increase athletic performance, and recent evidence has demonstrated an ergogenic effect of low and very low doses of caffeine taken late in prolonged exercise. Low caffeine doses do not alter exercise-induced changes in peripheral whole-body responses to exercise and are associated with few, if any, side effects. Low doses of caffeine (~200 mg) have also been shown to improve vigilance, alertness and mood, and improve cognitive processes during and following strenuous exercise. Therefore, the ergogenic effect of low caffeine doses appears to result from alterations in the CNS. However, many aspects of consuming low doses of caffeine remain unresolved and suffer from a lack of research. As the response to caffeine consumption is variable, athletes need to determine whether the ingestion of ~200 mg of caffeine before and/or during training and competitions is ergogenic on an individual basis.

## References

[1] Pasman WJ, VanBaak MA, Jeukendrup AE (1995). The effect of different dosages of caffeine on endurance performance time. Int J Sports Med..

[2] Graham TE, Spriet LL (1995). Metabolic, catecholamine and exercise performance responses to varying doses of caffeine. J Appl Physiol..

[3] Spriet LL, MacLean DA, Dyck DJ (1992). Caffeine ingestion and muscle metabolism during prolonged exercise in humans. Am J Physiol..

[4] Graham TE, Spriet LL (1991). Performance and metabolic responses to a high caffeine dose during prolonged exercise. J Appl Physiol..

[5] Graham TE, Helge JW, MacLean DA (2000). Caffeine ingestion does not alter carbohydrate or fat metabolism in skeletal muscle during exercise. J Physiol..

[6] Graham TE, Rush JWE, van Soeren MH (1994). Caffeine and exercise: metabolism and performance. Can J Appl Physiol..

[7] Spriet LL, Bahrke MS, Yesalis CE (2003). Caffeine. Performance enhancing substances in sport and exercise.

[8] Spriet LL. Caffeine. In: Maughan RJ, ed. The encyclopaedia of sports medicine: an IOC medical commission publication. Sports Nutrition. Vol 19. Oxford: Wiley; 2013. p. 313–23.

[9] Nehlig A, Daval JL, Debry G (1992). Caffeine and the central nervous system: mechanisms of action, biochemical, metabolic, and psychostimulant effects. Brain Res Rev..

[10] Daly JW, Garatttini S (1993). Mechanism of action of caffeine. Caffeine, coffee, and health.

[11] Fredholm BB (1995). Adenosine, adenosine receptors and the actions of caffeine. Pharmacol Toxicol..

[12] Kalmar JM, Cafarelli E (2004). Caffeine: a valuable tool to study central fatigue in humans. Exerc Sports Sci Rev..

[13] Davis JM, Zhao Z, Stock HS (2003). Central nervous system effects of caffeine and adenosine on fatigue. Am J Physiol..

[14] Graham TE, Hibbert E, Sathasivam P (1998). Metabolic and exercise endurance effects of coffee and caffeine ingestion. J Appl Physiol..

[15] Hodgson AB, Randell RK, Jeukendup AE (2013). The metabolic and performance effects of caffeine compared to coffee during endurance exercise. PLoS One.

[16] Kamimori GH, Karyekar CS, Otterstetter R (2002). The rate of absorption and relative bioavailability of caffeine administered in chewing gum versus capsules to normal healthy volunteers. Int J Pharm..

[17] Costill DL, Dalsky GP, Fink WJ (1978). Effects of caffeine on metabolism and exercise performance. Med Sci Sports..

[18] Ivy JL, Costill DL, Fink WJ (1979). Influence of caffeine and carbohydrate feedings on endurance performance. Med Sci Sports..

[19] Kovacs EMR, Stegen JHCH, Brouns F (1998). Effect of caffeine drinks on substrate metabolism, caffeine excretion, and performance. J Appl Physiol..

[20] Cox GR, Desbrow B, Montgomery PG (2002). Effect of different protocols of caffeine intake on metabolism and performance. J Appl Physiol..

[21] Talanian JL, Spriet LL (2007). Low doses of caffeine late in exercise improve cycling time trial performance. FASEB J.

[22] Jenkins NT, Trilk JL, Singhal A (2008). Ergogenic effects of low doses of caffeine on cycling performance. Int J Sport Nutr Exerc Metab..

[23] Desbrow B, Barrett CM, Minahan CL (2009). Caffeine, cycling performance, and exogenous CHO oxidation: a dose-response study. Med Sci Sports Exerc..

[24] Irwin C, Desbrow B, Ellis A (2011). Caffeine withdrawal and high-intensity endurance cycling performance. J Sports Sci..

[25] Desbrow B, Biddulph C, Devlin B (2012). The effects of different doses of caffeine on endurance cycling time trial performance. J Sports Sci..

[26] Burke LM (2008). Caffeine and sports performance. Appl Physiol Nutr Metab.

[27] Ganio MS, Klau JF, Casa DJ (2009). Effect of caffeine on sport-specific endurance performance: a systematic review. J Strength Cond Res..

[28] Wiles JD, Bird SR, Hopkins J (1992). Effect of caffeinated coffee on running speed, respiratory factors, blood lactate and perceived exertion during 1,500 m treadmill running. Br J Sports Med..

[29] Van Nieuwenhoven MA, Brouns F, Kovacs EM (2005). The effect of two sports drinks and water on GI complaints and performance during an 18 km run. Int J Sports Med..

[30] Bridge CA, Jones MA (2006). The effect of caffeine ingestion on 8 km run performance in a field setting. J Sports Sci..

[31] Schubert MM, Astorino TA, Azevedo JL (2013). The effects of caffeinated “energy shots” on time trial performance. Nutrients.

[32] Strecker E, Foster EB, Taylor K (2007). Effects of caffeine ingestion on tennis skill performance and hydration status [abstract]. Med Sci Sports Exerc.

[33] Stevenson EJ, Hayes PR, Allison SJ (2009). The effect of a carbohydrate-caffeine sports drink on simulated golf performance. Appl Physiol Nutr Metab..

[34] Perez-Lopez A, Salinero JJ, Abian-Vicen J, et al. Caffeinated energy drinks improve volleyball performance in elite female players. Med Sci Sports Exerc. Epub 18 Jul 2014. 10.1249/MSS.0000000000000455 25051390

[35] Del Coso J, Pereze-Lopez A, Abian-Vican L, et al. Caffeine-containing energy drink enhances physical performance in male volleyball players. Int J Sports Physiol Perform. Epub 19 Mar 2014. 10.1123/ijspp.2013-0448 24664858

[36] Gant N, Ali A, Foskett A (2010). The influence of caffeine and carbohydrate coingestion on simulated soccer performance. Int J Sports Nutr Exerc Metab..

[37] Roberts SP, Stokes KA, Trewartha G (2010). Effects of carbohydrate and caffeine ingestion on performance during a rugby union simulation protocol. J Sports Sci..

[38] Spriet LL, Hargreaves M, Spriet LL (2006). Anaerobic metabolism during exercise. Exercise metabolism.

[39] Parolin ML, Chesley A, Matsos MP (1999). Regulation of skeletal muscle glycogen phosphorylase and PDH during maximal intermittent exercise. Am J Physiol Endocrinol Metab..

[40] Astorino TA, Robertson DW (2010). Efficacy of acute caffeine ingestion for short-term high intensity exercise performance: a systematic review. J Strength Cond Res..

[41] Davis JK, Green JM (2009). Caffeine and anaerobic performance: ergogenic value and mechanisms of action. Sports Med..

[42] Jacobson BH, Weber MD, Claypool L (1992). Effect of caffeine on maximal strength and power in elite male athletes. Br J Sports Med..

[43] Woolf K, Bidwell W, Carlson AG (2008). The effect of caffeine as an ergogenic aid in anaerobic exercise. Int J Sport Nutr Exerc Metab..

[44] Wiles JD, Coleman D, Tegerdine M (2006). The effects of caffeine ingestion on performance time, speed and power during a laboratory-based 1-km cycling time trial. J Sports Sci..

[45] Doherty M, Smith P, Hughes M (2004). Caffeine lowers perceptual response and increases power output during high-intensity cycling. J Sports Sci..

[46] Anselme F, Collomp K, Mercier B (1992). Caffeine increases maximal anaerobic power and blood lactate concentration. Eur J Appl Occup Physiol..

[47] Collomp K, Ahmaidi S, Chatard JC (1992). Benefits of caffeine ingestion on sprint performance in trained and untrained swimmers. Eur J Appl Occup Physiol..

[48] Astorino TA, Terzi MN, Roberson DW (2010). Effect of two doses of caffeine on muscular function during isokinetic exercise. Med Sci Sports Exerc..

[49] Lieberman HR, Wurtman RJ, Emde GG (1987). The effects of low doses of caffeine on human performance and mood. Psychopharmacology..

[50] Olson CA, Thornton JA, Adam GE (2010). Effects of adenosine antagonists, quercitin and caffeine, on vigilance and mood. J Clin Pyschopharmacol..

[51] Brunye TT, Mahoney CR, Lieberman HR (2010). Caffeine modulates attention network function. Brain Cogn..

[52] Hogervorst E, Riedel WJ, Kovacs E (1999). Caffeine improves cognitive performance after strenuous physical exercise. Int J Sports Med..

[53] Hogervorst E, Badelow S, Scmitt J (2008). Caffeine improves physical and cognitive performance during exhaustive exercise. Med Sci Sports Exerc..

[54] Motl RW, OConnor PJ, Dishman RK (2003). Effect of caffeine on perceptions of leg muscle pain during moderate intensity cycling exercise. J Pain..

[55] Motl RW, OConnor PJ, Tubandt L (2006). Effect of caffeine on leg muscle pain during cycling exercise among females. Med Sci Sports Exerc.

[56] Gliottoni RC, Meyers JR, Arngrimsson SA (2009). Effect of caffeine on quadriceps muscle pain during acute cycling exercise in low versus high caffeine consumers. Int J Sport Nutr Exerc Metab..

[57] Astorino TA, Terzi MN, Roberson DW (2011). Effect of caffeine intake on pain perception during high intensity exercise. Int J Sport Nutr Exerc Metab..

[58] Ganio MS, Johnson EC, Lopez RM (2011). Caffeine lowers muscle pain during exercise in hot but not cool environments. Physiol Behav..

[59] Cohen BS, Nelson AG, Prevost MC (1996). Effects of caffeine ingestion on endurance racing in the heat and humidity. Eur J Appl Occup Physiol..

[60] Roelands B, Buyse L, Pauwels F (2011). No effect of caffeine on exercise performance in high ambient temperature. Eur J Appl Physiol..

[61] Ganio MS, Johnson EC, Klau JF (2011). Effect of ambient temperature on caffeine ergogenicity during endurance exercise. Eur J Appl Physiol..

[62] Van Nieuwenhoven MA, Brummer RJM, Brouns F (2000). Gastrointestinal function during exercise: comparison of water, sports drink, and sports drink with caffeine. J Appl Physiol..

[63] Yeo SE, Jentjens RLPG, Wallis GA (2005). Caffeine increases exogenous carbohydrate oxidation during exercise. J Appl Physiol.

[64] Hulston CJ, Jeukendrup AE (2008). Substrate metabolism and exercise performance with caffeine and carbohydrate intake. Med Sci Sports Exerc.

[65] Andoniou CE, Andrews DM, Degli-Esposti MA (2006). Natural killer cells in viral infection: more than just killers. Immunol Rev..

[66] Gleeson M (2007). Immune function in sport and exercise. J Appl Physiol.

[67] Fletcher DK, Bishop NC (2011). Effect of high and low dose of caffeine on antigen-stimulated activation of human natural killer cells after prolonged exercise. Int J Sport Nutr Exerc Metab..

[68] Syed SA, Kamimori GH, Kelly W (2005). Multiple dose pharmacokinetics of caffeine administered in chewing gum to normal healthy volunteers. Biopharm Drug Dispos..

[69] Ryan EJ, Kim CH, Muller MD (2012). Low-dose caffeine administered in chewing gum does not enhance cycling to exhaustion. J Strength Cond Res..

[70] Ryan EJ, Kim CH, Fickes EJ (2013). Caffeine gum and cycling performance: a timing study. J Strength Cond Res..

[71] Paton CD, Lowe T, Irvine A (2010). Caffeinated chewing gum increases repeated sprint performance and augments increases in testosterone in competitive cyclists. Eur J Appl Physiol..

[72] Bellar D, Kamimori G, Judge L (2012). Effects of low-dose caffeine supplementation on early morning performance in the standing shot put throw. Eur J Sports Sci..

[73] Doering TM, Fell JW, Veveritt MD (2014). The effect of a caffeinated mouth-rinse on endurance cycling time-trial performance. Int J Sport Nutr Exerc Metab..

[74] Beaven CM, Maulder P, Pooley A (2013). Effects of caffeine and carbohydrate mouth rinses on repeated sprint performance. Appl Physiol Nutr Metab..

